# Lessons learned from the pilot family model of diabetes self-management intervention in the Republic of the Marshall Islands

**DOI:** 10.1016/j.conctc.2023.101086

**Published:** 2023-02-06

**Authors:** Jennifer A. Andersen, Rachel S. Purvis, Aaron J. Scott, Joseph Henske, Dinesh Edem, James P. Selig, Jonell Hudson, Williamina Ioanna Bing, Jack Niedenthal, Henry Otuafi, Sheldon Riklon, Edlen Anzures, Ainrik George, Derek Alik, Pearl A. McElfish

**Affiliations:** a College of Medicine, University of Arkansas for Medical Sciences Northwest, Springdale, AR, 72762, USA; b Office of Community Health and Research, University of Arkansas for Medical Sciences Northwest, Springdale, AR, 72762, USA; c College of Medicine, University of Arkansas for Medical Sciences, Little Rock, AR, 72205, USA; d Fay W. Boozman College of Public Health, University of Arkansas for Medical Sciences Northwest, Springdale, AR, 72762, USA; e College of Pharmacy, University of Arkansas for Medical Sciences Northwest, Fayetteville, AR, 72703, USA; f Republic of the Marshall Islands Ministry of Health & Human Services, Majuro, MH, 96960, USA; g College of Medicine, University of Arkansas for Medical Sciences Northwest, Fayetteville, AR, 72703, USA

**Keywords:** Diabetes self-management education and support, Family model, Republic of the Marshall Islands, Type 2 diabetes, Intervention

## Abstract

**Background:**

The Republic of the Marshall Islands (RMI) has a high rate of type 2 diabetes mellitus (T2DM). To address the high rate of T2DM, we tested a culturally adapted family model of diabetes self-management education and support (F-DSMES). We report the results of the 12-month post-intervention data collection and describe the lessons learned from the delivery of the F-DSMES intervention.

**Methods:**

Recruitment took place in four churches in Majuro and included 10 h of content delivered over 8–10 weeks. Forty-one participants with T2DM were included. The primary study outcome was glycemic control measured by a change in HbA1c. We also conducted participant interviews to document the participant-reported barriers encountered during the F-DSMES intervention.

**Results:**

Participants did not show improvements in their biometric markers; however, participants did show improvement on multiple measures of diabetes knowledge and family support. We identified five areas to improve future interventions: 1) issues with recruitment, retention, and attendance; 2) needing help accessing information and additional healthcare provider counseling; 3) struggles with adhering to diet recommendations; 4) difficulty getting exercise, and 5) improving lessons within the intervention.

**Conclusion:**

Although the biomarker data did not show improvement, valuable information was gained to improve the development of larger-scale trials. The results provide evidence of the need for these trials and the desire of participants to continue pursuing this effort. Others doing similar work in other low-to-middle income countries will need to take into consideration the potential barriers and facilitators within participants’ social and physical environments.

## 1 Introduction

The Republic of the Marshall Islands (RMI) is an independent United States (US) Affiliated Pacific Islands nation [1]. Marshallese living in the Marshall Islands face numerous health disparities, including high prevalence rates of type 2 diabetes mellitus (T2DM) [[2], [3], [4], [5], [6], [7]]. Nearly a third of Marshallese living in the RMI have been diagnosed with T2DM [8], compared with lower rates in the US (11.3%) and globally (9.3%). These health disparities were likely perpetuated by historical trauma, including the nuclear weapons testing in the RMI by the US military throughout the 1940s and 1950s, which drastically altered the Marshallese lifestyle [9].

Socioecological barriers present in the RMI pose unique issues for the management of diabetes and other comorbidities. High rates of unemployment and a low minimum wage make it difficult to access fresh and nutritious foods [1]. Marshallese living in the RMI rely heavily on foods imported from the US, including rice and processed foods [12]. Gym memberships are cost-prohibitive for many in the RMI, and sidewalks are not widely available. Additionally, the infrastructure in the RMI limits the availability of health care and other resources, leaving many without a regular source of health care and posing difficulties obtaining diabetes testing supplies and medication [13].

Diabetes self-management education and support (DSMES) is critical for persons to effectively manage diabetes and reduce the risk of complications [[14], [15], [16], [17], [18]]. Yet, research regarding the use of DSMES in Marshallese and other Pacific Islander populations, especially among those living in the RMI, is limited [12,19,20]. Given the high rates of T2DM in the RMI, creating an effective DSMES intervention that can be widely implemented and disseminated is crucial.

In partnership with the local Marshallese community, the authors developed and tested a culturally tailored family model of DSMES (F-DSMES) in Arkansas [[21], [22], [23]]. Engagement in diabetes self-care is determined in large part by one's social environment; given the collectivist nature common in Marshallese culture, interventions with a focus on family support are part of culturally appropriate care. The F-DSMES intervention addressed diabetes management behaviors by focusing on behavioral changes in the context of family using motivational family interviewing, family goal-setting, and family education on supportive and non-supportive behaviors [[14], [15], [16], [17], [18], [19]].

In Arkansas, a trained bilingual community health worker (CHW) provided F-DSMES in the homes of people with diabetes [24,25]. A certified diabetes educator (CDE) attended all sessions. Compared to standard DSMES delivered by CDEs in a group setting, the F-DSMES demonstrated effectiveness when delivered in the home by CHWs [24,25]. However, the F-DSMES implementation methods are not easily transferred to the RMI [24,25]. The RMI has limited resources to deliver standard DSMES, and most homes in the RMI are too small to utilize for administering family education. Therefore, several important adaptations from the prior F-DSMES trial conducted in Arkansas needed to be introduced as part of the implementation of F-DSMES in the RMI. The F-DSMES intervention was delivered solely by trained CHWs, and the curriculum was delivered in faith-based organizations (FBOs) rather than in the home.

Our aim is to report the results of our pilot's 12 months post-intervention data, as well as to evaluate and document the participant-reported barriers encountered during the F-DSMES intervention. This pilot evaluation will be used to further improve future DSMES interventions in the RMI and to help to inform others who are doing similar work in other low-to-middle income countries.

## 2 Methods

### 2.1 Study aims and approach

The study's goal was to determine the acceptability, feasibility, and preliminary effectiveness of a culturally adapted and linguistically appropriate F-DSMES intervention.

### 2.2 Participant recruitment, enrollment, and consent

All materials and the study protocol were adapted from the materials developed as part of the Arkansas F-DSMES randomized controlled trial (RCT) (University of Arkansas for Medical Sciences [UAMS] Institutional Review Board [IRB] #203482) (Clinical Trial #NCT02407132) [24]. The pilot study was reviewed and approved by the UAMS IRB (#239272) and by the RMI Ministry of Health & Human Services [27].

Four churches on Majuro in the RMI agreed to participate in recruitment of study participants. Participants were required to meet inclusion criteria, including: (1) Marshallese, (2) ≥18 years, (3) a diagnosis of T2DM by a physician or a HbA1c indicative of diabetes (≥6.5%), (4) a family member in the same household willing to participate with the participant, and (5) willing to participate in all of the F-DSME sessions and in data collection. Participants were ineligible to participate in the study if they had participated in a DSMES program in the previous 5 years, if they planned to leave the area while the study was ongoing, or if they had any medical condition that the Data Safety and Monitoring team deemed would prevent successful completion of the program.

As recommended by the American Association of Diabetes Educators and the American Diabetes Association, the intervention included 10 h of education delivered over 8–10 weeks. Eight core elements are covered in the content: healthy diet, healthy levels of physical activity, monitoring blood glucose, understanding treatment of blood glucose and medications needs, problem-solving, risk management and coping skills, diabetes complications, and goal-setting [24]. One-hundred and twenty-five individuals were enrolled in the intervention. Of a total sample of 97, 41 participants had T2DM, and 56 family members agreed to participate. Further details on the study, including study design, recruitment, consent, inclusion and exclusion criteria, and intervention dosage, are available in prior publications [13,27,28].

### 2.3 Data collection

Research staff collected biometric data, including HbA1c, blood pressure, weight, and height measurements. HbA1c was collected via finger stick blood collection and point-of-care analyzer (Siemens DCA Vantage Analyzer) [27]. While the participant was seated, a research assistant used an OMRON digital blood pressure monitor to measure systolic and diastolic blood pressure. Pulse pressure was determined by subtracting the diastolic blood pressure reading from the systolic blood pressure reading. Participant height and weight were collected without shoes using a portable stadiometer and a digital scale. Body mass index (BMI) was calculated using weight in pounds and height in inches. Participants also completed a survey utilizing questions adapted from the Behavioral Risk Factor Surveillance System (BRFSS) survey's Diabetes and Healthcare Access Core Modules, as well as from the Diabetes Care Profile [24,29]. Participants could refuse to participate in any part of the data collection and still participate in the F-DSMES educational intervention. Participants were given their results from the health screening, as well as private health counseling and a referral to a healthcare provider if warranted.

To capture lessons learned to inform a larger RCT of the F-DSMES intervention in the RMI, the research staff conducted interviews with eight participants. Bilingual (Marshallese and English) study staff trained in qualitative methods facilitated the focus groups, and participants were encouraged to speak in their preferred language. The interviews allowed participants to describe their experiences; topics explored included facilitators and barriers to participation, parts of the intervention the participants found useful, and where they saw need for improvement.

### 2.4 Analytical methods

*Quantitative analysis.* The mean and standard deviation for continuous variables and proportions for categorical variables are provided for participant sociodemographic data, biometric data, and self-reported health status compared to 12 months ago. In addition, we provide descriptive statistics of participants' diabetes management and diabetes knowledge, barriers to obtaining care, and family support. Wilcoxon Ranked Sign tests were used for mean comparisons, and McNemar's tests and Bowkers [30] tests of symmetry were used for the comparisons of counts or proportions. Although the small sample size of the study limits the strength of the conclusions from the hypothesis tests, it allows for assessment of trends that could be anticipated to result in a meaningful change in clinically relevant outcomes. In addition, we include an assessment of individual participant changes in the outcomes of interest. This assessment allows us to examine whether aggregate changes were consistent across participants or whether they were magnified by one or more outliers. All analyses were conducted using SAS 9.4 [31].

*Qualitative analysis.* All interviews were audio recorded, transcribed, and translated verbatim into English by bilingual study staff. The content analysis, conducted by the lead and second author, was completed by reviewing and manually coding the interview transcripts to interpret meaning and assign labels to data segments with initial codes [[32], [33], [34]]. Initial coding was conducted by the first author, labeling data segments with concise summary codes in order to organize the data for more focused coding and to develop a preliminary codebook of emergent themes. Confirmation coding on the transcripts was completed by the second author, and the research team reviewed the coded interview transcripts together to categorize the data and develop themes [34,35]. The research team discussed any discrepancies; all discrepancies were resolved by consensus. The most illustrative quotes for each theme were identified and are presented.

## 3 Results

### 3.1 Quantitative results

Table 1 reports the sociodemographic characteristics of the participants at baseline and at 12-month follow-up. Twenty-eight of the original 41 baseline participants returned for the 12-month follow-up visit. The mean age of the participants at the 12-month follow-up was 52.6 years (SD = 13.1). Among the participants in the 12-month follow-up sample, 82.1% were females, and 71.4% were married or a member of an unmarried couple. The majority of participants (60.7%) had less than a high school education, and approximately half (53.6%) reported speaking English well or very well. The participants completed a mean of 5.4 (±3.6) h of education classes; 17% (7) completed 8–10 h, 34% (14) completed 5–7 h, and 49% (20) completed four or less hours of the intervention, as reported in a previous publication [13].

**Table 1:** Sociodemographic characteristics of primary participants – Baseline and 12-month follow-up.

	Baseline (N = 41)	12 Months (N = 28)
	*Mean* ± *SD or n(%)*	*Mean* ± *SD or n(%)*
Age (in years)	51.3± 12.4	52.6 ± 13.1
Sex (Female)	31(73.8)	23(82.1)
Marital Status		
Married/Member of unmarried couple	32(76.2)	20(71.4)
Single/Divorced/Widowed	10(23.8)	8(28.6)
Education		
Less than HS diploma	30(66.7)	17(60.7)
HS diploma/Some college	13(31.0)	10(35.7)
College degree or more	1(2.4)	1(3.6)
Speak English		
Very well/Well	19(45.2)	15(53.6)
Not well/Not at all	23(54.8)	13(46.4)

Table 2 presents the health profiles of the participants with both baseline and 12-month post-intervention data. Changes in health measures were not statistically significant. Participants had a mean increase in HbA1c of 0.6% (p = .089) and a mean decrease in BMI of 0.7 kg/m2 (p = .431). Random blood glucose showed a mean increase of 23.4 mg/dL (p = .107). There was a mean increase in total cholesterol of 6.5 mg/dL (p = .617), a mean increase in triglycerides of 14.4 mg/dL (p = .558), a mean decrease in low-density lipoprotein (LDL) of 0.9 mg/dL (p = .528), and a mean increase in high-density lipoprotein (HDL) of 1.2 mg/dL (p = .192). Participants had a mean decrease in diastolic and systolic blood pressure of 4.1 mmHg (p = .142) and 4.2 mmHg (p = .676), respectively. There was no mean change in pulse pressure, self-reported health status, or health status compare to 12 months prior.

**Table 2:** Health profiles of study participants – Baseline and 12-month follow-up comparison.

	Baseline (n = 28)		12 Months (n = 28)		*p*^a^
	*Mean* ± *SD or n(%)*	*Median*	*Mean* ± *SD or n(%)*	*Median*	
**Blood Glucose**					
**HbA1c**	10.2 ± 2.3	10.7	10.8 ± 2.5	11.2	0.089
**Random glucose**	203.6 ± 85.6	198.5	227.0 ± 78.2	218.0	0.107
**Weight**					
**Weight (in lbs.)**	162.9 ± 35.4	162.6	158.8 ± 33.9	155.2	0.212
**BMI**	31.3 ± 6.0	6.0	30.6 ± 5.9	30.5	0.431
**BMI** < **30**	12(42.9)	–	12(42.9)	–	1.000
**BMI** ≥ **30**	16(57.1)	–	16(57.1)	–	
**Blood Pressure**					
**Diastolic**	77.1 ± 11.4	77.0	73.0 ± 10.6	75.0	0.142
**Systolic**	124.3 ± 18.2	121.0	120.1 ± 14.8	121.0	0.676
**Pulse pressure**	47.1 ± 15.6	45.0	47.1 ± 12.2)	46.0	0.934
**Cholesterol Levels**					
**HDL**	32.8 ± 9.5	9.5	34.0 ± 6.6	35.0	0.192
**LDL**	111.7 ± 22.9	113.5	110.8 ± 28.3	111.5	0.528
**Triglycerides**	135.0 ± 52.0	131.0	149.4 ± 88.7	128.0	0.558
**Total cholesterol**	166.4 ± 27.8	164.0	172.9 ± 28.8	171.0	0.617
**Self-Rated Health**					
**Current Health Status**		1.000			
**Excellent/Good**	10(35.7)	–	10(35.7)	–	
**Fair/Poor**	18(64.3)	–	18(64.3)	–	
**Current Health Status Compared to 12 Months Ago**		0.481			
**Better**	8(28.6)	–	12(42.9)	–	
**About the same**	14(50.0)	–	12(42.9)	–	
**Worse**	6(21.4)	–	4(14.3)	–	

*Note:* Sample sizes vary due to missing data; BMI and weight missing values are due to participant limitations.

a Wilcoxon Ranked Sign test for mean comparisons, McNemar's & Bowkers [30] test of symmetry for counts/proportions.

Disaggregated results for participant changes in HbA1c, random glucose testing, blood pressure, HDL cholesterol, and LDL cholesterol from baseline to 12-month post-intervention are provided in the supplemental tables. For HbA1c, 11 of the participants showed improvements from baseline to 12-month post-intervention, while one showed no change and 15 had higher HbA1c results. Improvements ranged from 0.1% to 2.0%, and seven of the 11 participants had improvements of 0.5% or higher. Increases in HbA1c ranged from 0.3% to 5.3%, with 13 of the 15 participants experiencing increases in HbA1c of 0.5% or higher. Eight of the participants showed improved random glucose numbers, ranging from a decrease of 1 mg/dL to 219 mg/dL. Twenty participants had an increase in random glucose numbers, ranging from 4 mg/dL to 170 mg/dL. Fourteen of the participants had an improved systolic blood pressure, with a decrease ranging between 2 mmHg and 47 mmHg. Fifteen participants had an improved diastolic blood pressure, with a decrease between 2 mmHg and 33 mmHg. Fifteen participants had an improvement in HDL, ranging between 1 mg/dL and 18 mg/dL. Three participants had no change in HDL. Fourteen participants had an improvement in LDL, with a decrease between 3 mg/dL and 52 mg/dL.

Participants showed statistically significant improvement in their diabetes knowledge at 12-month follow-up (Table 3). Participants reported gained knowledge on how to manage their diabetes (p = .005) and cope with stress (p = .017), as well as a better understanding of the role of exercise in diabetes care (p = .007), how diet, exercise, and medications affect blood sugar levels (p = .006), and how to take their medications correctly (p = .038). Participants also reported increased knowledge of how to treat high (p = .010) and low (p = .019) blood sugar, how to prevent long-term complications of diabetes (p = .006), how to take care of their feet (p = .016), and the benefits of improving blood sugar control (p = .007). Reported changes in knowledge related to how to eat for blood sugar control (p = .059) or how to use the results of blood sugar monitoring (p = .372) were not significant. Participants reported statistically significant improved family support for taking their diabetes medicine (p = .029), for taking care of their feet (p = .015), and in handling their feelings about their diabetes (p = .038); however, no statistically different change in reported family support was found for following a meal plan (p = .518), getting enough physical activity (p = .308), or testing blood sugar (p = .287; see Table 4).

**Table 3:** Diabetes knowledge – Baseline and 12-month follow-up comparison among participants with prior diabetes diagnosis (*n* = *22*).

	Baseline	12 Months	*p*^a^
	*n(%)*	*n(%)*	
***How well do you understand … ?***			
**How to manage your diabetes?**			**0.005**^b^
Not at all	1(4.6)	1(4.6)	
A little	21(95.5)	9(40.9)	
A lot	0(0.0)	12(54.6)	
**How to cope with stress?**			**0.017**
Not at all	5(22.7)	2(9.1)	
A little	15(68.2)	8(36.4)	
A lot	2(9.1)	12(54.6)	
**How to eat for blood sugar control?**			0.059
Not at all	4(18.2)	2(9.1)	
A little	17(77.3)	10(45.5)	
A lot	1(4.6)	10(45.5)	
**The role of exercise in diabetes care?**			**0.007**
Not at all	2(9.1)	1(4.6)	
A little	18(81.8)	7(31.8)	
A lot	2(9.1)	14(63.6)	
**How to take your medications correctly?**			**0.038**
Not at all	4(18.2)	1(4.6)	
A little	11(50.0)	5(22.7)	
A lot	7(31.8)	16(72.7)	
**How to use the results of blood sugar monitoring?**			0.372
Not at all	5(22.7)	2(9.1)	
A little	8(36.4)	7(31.8)	
A lot	9(40.9)	13(59.1)	
**How diet, exercise, and medications affect blood sugar levels?**			**0.006**
Not at all	3(13.6)	2(9.1)	
A little	18(81.8)	7(31.8)	
A lot	1(4.6)	13(59.1)	
**How to prevent and treat high blood sugar?**			**0.010**^b^
Not at all	5(22.7)	2(9.1)	
A little	17(77.3)	9(40.9)	
A lot	0(0.0)	11(50.0)	
**How to prevent and treat low blood sugar?**			**0.019**
Not at all	6(27.3)	2(9.1)	
A little	14(63.6)	8(36.4)	
A lot	2(9.1)	12(54.2)	
**How to prevent long-term complications of diabetes?**			**0.006**
Not at all	5(22.7)	2(9.1)	
A little	16(72.7)	7(31.8)	
A lot	1(4.6)	13(59.1)	
**How to take care of your feet?**			**0.016**
Not at all	4(18.2)	1(4.6)	
A little	14(63.6)	5(22.7)	
A lot	4(18.2)	16(72.7)	
**The benefits of improving blood sugar control?**			**0.007**
Not at all	3(13.6)	1(4.6)	
A little	17(77.3)	8(36.4)	
A lot	2(9.1)	13(59.1)	

a Bowker's test of symmetry [30].

b Weighted count, accounting for zero(s).

**Table 4:** Family support for diabetes management – Baseline and 12-month follow-up comparison among participants with prior diabetes diagnosis (*n* = *22*).

	Baseline	12 Months	*p*^a^
	*n(%)*	*n(%)*	
***My family helps me to … ?***			
**Follow my meal plan**			0.518
Not at all	1(4.6)	1(4.6)	
A little	10(45.5)	5(22.7)	
A lot	11(50.0)	16(72.7)	
**Take my medicine**			**0.029**
Not at all	2(9.1)	2(9.1)	
A little	11(50.0	2(9.1)	
A lot	9(40.9)	18(81.8)	
**Take care of my feet**			**0.015**
Not at all	6(27.3)	1(4.6)	
A little	10(45.5)	4(18.2)	
A lot	6(27.3)	17(77.3)	
**Get enough physical activity**			0.308
Not at all	1(4.8)	1(4.8)	
A little	10(47.6)	4(19.1)	
A lot	10(47.6)	16(76.2)	
**Test my blood sugar**			0.287
Not at all	2(9.1)	1(4.6)	
A little	10(45.5)	5(22.7)	
A lot	10(45.5)	16(72.7)	
**Handle my feelings about diabetes**			**0.038**
Not at all	1(4.6)	1(4.6)	
A little	13(59.1)	4(18.2)	
A lot	8(36.6)	17(77.3)	

a Bowker's test of symmetry [30].

### 3.2 Qualitative results

Participants discussed their experiences with the intervention in qualitative interviews. The research team identified five themes from participants' experiences related to the acceptability and feasibility study: 1) issues with recruitment, retention, and attendance; 2) needing help accessing information and additional healthcare provider counseling; 3) struggles with adhering to diet recommendations; 4) difficulty getting exercise, and 5) improving lessons within the intervention.

*Issues with recruitment, retention, and attendance.* Participants noted there were many people who were interested in classes but did not attend church services, or attended services at churches who were not part of the recruitment and were unsure if they could participate. Participants said recruitment should be more widespread, additional churches should be included in the recruitment process, and placing flyers at other local establishments (e.g., stores) would increase the number of people who would participate. Participants stated class schedules were difficult to remember, making it difficult to attend. Participants did stop attending classes and data collection events over time. A participant explained: “I did ask other people who stopped attending the classes, and they said they stopped because their family members stopped attending. I told them even if other family members stopped attending, they should keep attending.”

*Needing help accessing information and additional healthcare provider counseling.* Participants requested that additional information on their health conditions be in the lessons. Participants also requested additional help with accessing physicians to discuss medications such as insulin, as well as diabetes comorbidities such as hypercholesterolemia: “We wanted to have diabetes doctors at screenings to provide more direct care at the churches. Many of us didn't know we had high cholesterol, or high blood pressure, or blood sugar, until you had the screenings. So provide more personal direct counseling to screenings.”

*Struggles with adhering to diet recommendations.* Participants reported struggling with diet changes and adhering to dietary recommendations. Vegetables are costly and hard to access in the RMI, and rice is a staple in the Marshallese diet. Participants felt rice helped with satiation, making it difficult to limit portion sizes and feel as though they had enough to eat. For example, one participant noted, “We just couldn't eat vegetables.” She went on to say, “We couldn't seem to get full or satisfied on a small amount of rice! There needs to be plenty of rice.” Additionally, the use of a portion plate was difficult because of both the lack of food options and traditional meals being different from what the program taught. One participant stated, “The portion plate was sort of difficult because we were so used to having like 3 scoops of rice, but now we have to reduce that!”

*Difficulty getting exercise.* Participants noted getting exercise was difficult for various reasons, including a lack of good walking shoes, limited access to walking areas, and the cost of gym memberships. One participant described their experiences with attempting to get more exercise:

Sometimes I walk over to the Mormon Church and walk around the area and exercise. I tried to exercise often but I don't have good walking shoes. I had walking shoes that my son send from the Hawaii. The shoes were really nice and light. My neighbor's sister borrowed my shoes, but when she moved, she never brought them back. I also exercise outside my house, but now there's construction as you can see.

*Improving lessons within the intervention.* Participants stated they enjoyed the lessons and found them informative and not too long; however, participants also mentioned needing more explanations or demonstrations incorporated into lesson plans. For example, participants said, “Only more demonstrations because we really enjoyed the classes that had the demonstrations like the foot model and such,” and “Topic or lessons were good, just provide more details or spend more time explaining lessons.”

## 4 Discussion

Participants did not show statistically significant improvements in their biometric markers. Research has shown that improvements in diabetes knowledge and family support have the ability to improve health outcomes, including HbA1c [36,37]. However, despite the improvement in most knowledge domains and some family support domains, we did not see an improvement in HbA1c. The socioecological barriers in the RMI may make it more difficult to apply the knowledge and support gained through the F-DSMES intervention. The socioecological barriers in the RMI are numerous. Unemployment hovers around 40%, and the national minimum wage is low, making fresh, healthy foods difficult to obtain due to cost [1]. As participants noted in their interviews, Marshallese living in the RMI rely heavily on cheaper, and what they perceive as more filling, options such as rice and processed foods. Many Marshallese living in the RMI find fresh, unprocessed foods cost-prohibitive [12]. Further, participants stated they have a lack of access to good footwear and that roads lack sidewalks, making exercise difficult. Further, prior work has shown many Marshallese living in the RMI do not have a regular source of care to go to for information on their health and often report difficulties affording diabetes testing supplies and medication [13]. The immense number of socioecological barriers makes managing diabetes and other comorbidities difficult and may have contributed to the lack of improvement seen in the results both for the biometric measures and in the few areas where knowledge and family support did not increase (meal planning, exercise, and using blood sugar results).

There are a few limitations to keep in mind when interpreting these results. Participants have very limited access to healthcare services; therefore, we are unable to obtain a complete list of pre-existing medical conditions which may affect biometric readings (e.g., anemia and HbA1c). The F-DSMES intervention struggled with retention and attendance. Nearly a third of the participants were lost to follow-up. The high attrition may limit the generalizability to the RMI as a whole, as well as to other locations. Further, as only eight participants were interviewed, we may not have a full picture of the barriers to participation experienced. Despite these limitations, the participants did provide us with valuable insight to adapt the F-DSMES intervention used in the RMI, which will likely improve the results.

### 4.1 Future directions

In future trials of the F-DSMES intervention in the RMI, we plan to work to address the barriers to recruitment, attendance, and retention as well as the socioecological barriers, which make controlling diabetes more difficult. These efforts will be directed by the suggestions made by the participants during their interviews and our community advisory board. We plan to expand recruitment efforts to additional locations. We will design a reminder card to remind participants of the upcoming classes, with a place to make notes if a schedule does change.

Although bringing physicians and other care providers to in-person screenings and lessons may be difficult, we will work with the RMI Ministry of Health & Human Services to have additional healthcare providers on site whenever possible, and we are exploring the potential for Marshallese care providers to host ‘ask me anything’ sessions using Zoom/telemedicine. Marshallese providers could be based in the RMI and/or in US to answer more general questions related to diabetes and other comorbidities. Additionally, we will work with RMI-based CHWs to provide care navigation to providers in the RMI.

Further, we will work to assist participants in meeting their diet and exercise recommendations. This will include additional work on creating healthy meals that meet nutrition goals using only available and traditional foods, providing healthy food boxes, and/or providing opportunities for participants and communities to create raised gardens to supplement the available fruit and vegetable supply. We will work toward supplying appropriate walking shoes and will collaborate with the appropriate community-based organizations to create resources for both group-based and individual exercise.

Finally, in order to adapt the F-DSMES to meet the needs of Marshallese living in the RMI, we will make the F-DSMES program more interactive and spend more time incorporating anatomical models and demonstrations into the curriculum.

## 5 Conclusion

This study is one of the first to provide culturally appropriate care for people with diabetes living in the RMI by implementing a family-based diabetes self-management and education program within faith-based organizations (churches). In addition, it is the first to outline participant feedback on the intervention. Although the biomarker data did not show improvement, we have gained valuable information to incorporate in the development of larger scale trials and have provided evidence of both the need for these trials and the desire of participants to continue pursuing this effort. We urge others who are doing similar work in other low-to-middle income countries to take into consideration the potential barriers and facilitators within participants’ social and physical environments and to work towards ensuring that participants have access to the resources and information they need to successfully navigate barriers present in the RMI.

## Funding

This study was made possible because of a community-based participatory research partnership with local Marshallese faith-based leaders, the RMI Ministry of Health & Human Services, Kora In Jiban Lolorjake Ejmour (KIJLE), and the Marshallese Consulate General in Springdale, Arkansas. Community engagement efforts were supported by 10.13039/100008519University of Arkansas for Medical Sciences Translational Research Institute funding awarded through the 10.13039/100006108National Center for Advancing Translational Sciences of the 10.13039/100000002National Institutes of Health (number 1U54TR001629-01A1). An award from the Sturgis Foundation supported the diabetes self-management education pilot study. Dr. Andersen was supported by University of Arkansas for Medical Sciences Translational Research Institute funding awarded through the 10.13039/100006108National Center for Advancing Translational Sciences of the National Institutes of Health (number KL2TR003108). The content is solely the responsibility of the authors and does not necessarily represent the official views of the National Institutes of Health.

## Data availability

The deidentified data underlying the results presented in this study may be made available upon reasonable request from the corresponding author, Dr. Pearl A. McElfish, at pamcelfish@uams.edu.

## Declaration of competing interest

The authors declare that they have no known competing financial interests or personal relationships that could have appeared to influence the work reported in this paper.
