# Supplementary material for: Lessons learned from the pilot family model of diabetes self-management intervention in the Republic of the Marshall Islands
Source: Contemp Clin Trials Commun. 2023 Feb 6;32:101086. doi: 10.1016/j.conctc.2023.101086 (PMC9929673; doi:10.1016/j.conctc.2023.101086)
Supplement: Multimedia component 1 [file mmc1.docx]

**Supplemental Table 1.** Baseline, 12-month follow-up, and change scores of HbA1c and random glucose test results for primary participants

|  | HbA1c | | | | |  | | | Random Glucose | | |  |  |
| --- | --- | --- | --- | --- | --- | --- | --- | --- | --- | --- | --- | --- | --- |
|  | Baseline | 12 Months | | Change | | |  | | Baseline | 12 Months | Change | |  |
| 1. Participant 1 | | 11.5 | | 10.1 | | **-1.4** | | | 1. Participant 1 | 243 | 263 | 20 | |
| 1. Participant 2 | | 6.7 | | 8.6 | | 1.9 | | | 1. Participant 2 | 98 | 154 | 56 | |
| 1. Participant 3 | | 14.0 | | 13.0 | | **-1.0** | | | 1. Participant 3 | 437 | 218 | **-219** | |
| 1. Participant 5 | | 11.2 | | 13.7 | | 2.5 | | | 1. Participant 5 | 230 | 357 | 127 | |
| 1. Participant 10 | | 8.9 | | 7.8 | | **-1.1** | | | 1. Participant 10 | 229 | 167 | **-62** | |
| 1. Participant 13 | | 7.7 | | 10.1 | | 2.4 | | | 1. Participant 13 | 99 | 236 | 137 | |
| 1. Participant 18 | | 6.7 | | 6.7 | | 0.0 | | | 1. Participant 18 | 115 | 159 | 44 | |
| 1. Participant 24 | | 6.5 | | 6.4 | | **-0.1** | | | 1. Participant 24 | 96 | 100 | 4 | |
| 1. Participant 27 | | 6.5 | | 7.0 | | 0.5 | | | 1. Participant 27 | 121 | 120 | **-1** | |
| 1. Participant 29 | | 10.2 | | 11.2 | | 1.0 | | | 1. Participant 29 | 164 | 175 | 11 | |
| 1. Participant 31 | | 11.5 | | 9.7 | | **-1.8** | | | 1. Participant 31 | 242 | 204 | **-38** | |
| 1. Participant 32 | | 10.7 | | 10.6 | | **-0.1** | | | 1. Participant 32 | 176 | 251 | 75 | |
| 1. Participant 35 | | 11.0 | | 11.8 | | 0.8 | | | 1. Participant 35 | 261 | 418 | 157 | |
| 1. Participant 36 | | 10.0 | | 14.0 | | 4.0 | | | 1. Participant 36 | 290 | 315 | 25 | |
| 1. Participant 39 | | 7.6 | | 9.9 | | 2.3 | | | 1. Participant 39 | 101 | 190 | 89 | |
| 1. Participant 40 | | 12.6 | | 12.5 | | **-0.1** | | | 1. Participant 40 | 273 | 281 | 8 | |
| 1. Participant 45 | | 13.6 | | 11.6 | | **-2.0** | | | 1. Participant 45 | 192 | 332 | 140 | |
| 1. Participant 47 | | 8.8 | | 14.1 | | 5.3 | | | 1. Participant 47 | 134 | 247 | 113 | |
| 1. Participant 51 | | 8.9 | | 11.3 | | 2.4 | | | 1. Participant 51 | 204 | 218 | 14 | |
| 1. Participant 52 | | 11.5 | | 12.0 | | 0.5 | | | 1. Participant 52 | 146 | 153 | 7 | |
| 1. Participant 55 | | 11.5 | | 13.7 | | 2.2 | | | 1. Participant 55 | 114 | 284 | 170 | |
| 1. Participant 56 | | 9.8 | | 10.2 | | 0.4 | | | 1. Participant 56 | 243 | 209 | **-34** | |
| 1. Participant 60 | | 11.4 | | 14.1 | | 2.7 | | | 1. Participant 60 | 252 | 343 | 91 | |
| 1. Participant 62 | | 12.8 | | 12.6 | | **-0.2** | | | 1. Participant 62 | 315 | 242 | **-73** | |
| 1. Participant 64 | | 13.8 | | 14.1 | | 0.3 | | | 1. Participant 63 | 356 | 214 | **-142** | |
| 1. Participant 67 | | 7.5 | | 6.3 | | **-1.2** | | | 1. Participant 65 | 193 | 233 | 40 | |
| 1. Participant 69 | | 11.3 | | 9.6 | | **-1.7** | | | 1. Participant 68 | 134 | 103 | **-31** | |
| **Bolded** difference scores indicate improvement. | | | | | | | | | 1. Participant 70 | 242 | 169 | **-73** | |

**Supplemental Table 2.** Baseline, 12-month follow-up, and change scores of systolic and diastolic blood pressure for primary participants

|  | Systolic BP | | | | |  | | | Diastolic BP | | |  |  |
| --- | --- | --- | --- | --- | --- | --- | --- | --- | --- | --- | --- | --- | --- |
|  | Baseline | 12 Months | | Change | | |  | | Baseline | 12 Months | Change | |  |
| 1. Participant 1 | | 105.0 | | 105.0 | | 0.0 | | | 1. Participant 1 | 72.0 | 63.0 | **-9.0** | |
| 1. Participant 2 | | 111.0 | | 124.0 | | 13.0 | | | 1. Participant 2 | 59.0 | 78.0 | 19.0 | |
| 1. Participant 3 | | 157.0 | | 115.0 | | **-42.0** | | | 1. Participant 3 | 91.0 | 69.0 | **-22.0** | |
| 1. Participant 5 | | 109.0 | | 131.0 | | 22.0 | | | 1. Participant 5 | 70.0 | 78.0 | 8.0 | |
| 1. Participant 10 | | 110.0 | | 104.0 | | **-6.0** | | | 1. Participant 10 | 58.0 | 61.0 | 3.0 | |
| 1. Participant 13 | | 130.0 | | 120.0 | | **-10.0** | | | 1. Participant 13 | 81.0 | 72.0 | **-9.0** | |
| 1. Participant 18 | | 99.0 | | 105.0 | | 6.0 | | | 1. Participant 18 | 68.0 | 58.0 | **-10.0** | |
| 1. Participant 24 | | 145.0 | | 154.0 | | 9.0 | | | 1. Participant 24 | 52.0 | 61.0 | 9.0 | |
| 1. Participant 27 | | 110.0 | | 122.0 | | 12.0 | | | 1. Participant 27 | 65.0 | 74.0 | 9.0 | |
| 1. Participant 29 | | 123.0 | | 123.0 | | 0.0 | | | 1. Participant 29 | 77.0 | 79.0 | 2.0 | |
| 1. Participant 32 | | 120.0 | | 128.0 | | 8.0 | | | 1. Participant 32 | 77.0 | 80.0 | 3.0 | |
| 1. Participant 33 | | 121.0 | | 95.0 | | **-26.0** | | | 1. Participant 33 | 73.0 | 40.0 | **-33.0** | |
| 1. Participant 36 | | 122.0 | | 118.0 | | **-4.0** | | | 1. Participant 36 | 78.0 | 70.0 | **-8.0** | |
| 1. Participant 37 | | 172.0 | | 125.0 | | **-47.0** | | | 1. Participant 37 | 93.0 | 75.0 | **-18.0** | |
| 1. Participant 40 | | 110.0 | | 100.0 | | **-10.0** | | | 1. Participant 40 | 72.0 | 64.0 | **-8.0** | |
| 1. Participant 41 | | 119.0 | | 110.0 | | **-9.0** | | | 1. Participant 41 | 87.0 | 79.0 | **-8.0** | |
| 1. Participant 46 | | 125.0 | | 99.0 | | **-26.0** | | | 1. Participant 46 | 75.0 | 65.0 | **-10.0** | |
| 1. Participant 48 | | 146.0 | | 144.0 | | **-2.0** | | | 1. Participant 48 | 81.0 | 79.0 | **-2.0** | |
| 1. Participant 52 | | 140.0 | | 127.0 | | **-13.0** | | | 1. Participant 52 | 81.0 | 79.0 | **-2.0** | |
| 1. Participant 53 | | 113.0 | | 137.0 | | 24.0 | | | 1. Participant 53 | 88.0 | 79.0 | **-9.0** | |
| 1. Participant 56 | | 109.0 | | 123.0 | | 14.0 | | | 1. Participant 56 | 75.0 | 79.0 | 4.0 | |
| 1. Participant 57 | | 111.0 | | 121.0 | | 10.0 | | | 1. Participant 57 | 71.0 | 81.0 | 10.0 | |
| 1. Participant 61 | | 130.0 | | 144.0 | | 14.0 | | | 1. Participant 61 | 83.0 | 89.0 | 6.0 | |
| 1. Participant 63 | | 122.0 | | 133.0 | | 11.0 | | | 1. Participant 63 | 89.0 | 93.0 | 4.0 | |
| 1. Participant 64 | | 112.0 | | 104.0 | | **-8.0** | | | 1. Participant 64 | 73.0 | 75.0 | 2.0 | |
| 1. Participant 66 | | 124.0 | | 118.0 | | **-6.0** | | | 1. Participant 66 | 94.0 | 77.0 | **-17.0** | |
| 1. Participant 70 | | 160.0 | | 113.0 | | **-47.0** | | | 1. Participant 70 | 99.0 | 73.0 | **-26.0** | |
| 1. Participant 71 | | 204.2 | | 216.4 | | 12.2 | | | **Bolded** difference scores indicate improvement. | | | | |

**Supplemental Table 3.** Baseline, 12-month follow-up, and change scores of HDL and LDL for primary participants

|  | HDL | | | | |  | | | LDL | | |  |  |
| --- | --- | --- | --- | --- | --- | --- | --- | --- | --- | --- | --- | --- | --- |
|  | Baseline | 12 Months | | Change | | |  | | Baseline | 12 Months | Change | |  |
| 1. Participant 1 | | 39 | | 39 | | **0** | | | 1. Participant 1 | 113 | 77 | **-36** | |
| 1. Participant 2 | | 22 | | 30 | | **8** | | | 1. Participant 2 | 99 | 88 | **-11** | |
| 1. Participant 3 | | 22 | | 22 | | **0** | | | 1. Participant 3 | 124 | 96 | **-28** | |
| 1. Participant 5 | | 26 | | 26 | | **0** | | | 1. Participant 4 | 86 | 101 | 15 | |
| 1. Participant 10 | | 24 | | 23 | | -1 | | | 1. Participant 9 | 118 | 90 | **-28** | |
| 1. Participant 13 | | 41 | | 35 | | -6 | | | 1. Participant 12 | 118 | 114 | **-4** | |
| 1. Participant 18 | | 43 | | 44 | | 1 | | | 1. Participant 15 | 108 | 56 | **-52** | |
| 1. Participant 24 | | 38 | | 35 | | -3 | | | 1. Participant 20 | 157 | 163 | 6 | |
| 1. Participant 27 | | 46 | | 39 | | -7 | | | 1. Participant 23 | 96 | 86 | **-10** | |
| 1. Participant 29 | | 24 | | 23 | | -1 | | | 1. Participant 26 | 101 | 91 | **-10** | |
| 1. Participant 31 | | 20 | | 26 | | **6** | | | 1. Participant 27 | 140 | 107 | **-33** | |
| 1. Participant 32 | | 42 | | 39 | | -3 | | | 1. Participant 30 | 127 | 134 | 7 | |
| 1. Participant 35 | | 43 | | 31 | | -12 | | | 1. Participant 31 | 130 | 116 | **-14** | |
| 1. Participant 36 | | 34 | | 38 | | **4** | | | 1. Participant 34 | 114 | 120 | 6 | |
| 1. Participant 39 | | 38 | | 37 | | -1 | | | 1. Participant 39 | 124 | 150 | 26 | |
| 1. Participant 40 | | 29 | | 30 | | **1** | | | 1. Participant 41 | 88 | 143 | 55 | |
| 1. Participant 45 | | 39 | | 41 | | **2** | | | 1. Participant 45 | 67 | 144 | 77 | |
| 1. Participant 47 | | 27 | | 35 | | **8** | | | 1. Participant 46 | 82 | 67 | **-15** | |
| 1. Participant 51 | | 26 | | 35 | | **9** | | | 1. Participant 49 | 86 | 94 | 8 | |
| 1. Participant 52 | | 62 | | 41 | | -21 | | | 1. Participant 50 | 142 | 145 | 3 | |
| 1. Participant 55 | | 26 | | 28 | | **2** | | | 1. Participant 54 | 86 | 109 | 23 | |
| 1. Participant 56 | | 30 | | 33 | | **3** | | | 1. Participant 56 | 142 | 133 | **-9** | |
| 1. Participant 60 | | 26 | | 34 | | **8** | | | 1. Participant 57 | 105 | 123 | 18 | |
| 1. Participant 62 | | 23 | | 41 | | **18** | | | 1. Participant 59 | 145 | 142 | **-3** | |
| 1. Participant 63 | | 33 | | 37 | | **4** | | | 1. Participant 62 | 114 | 120 | 6 | |
| 1. Participant 65 | | 31 | | 32 | | **1** | | | 1. Participant 64 | 92 | 72 | **-20** | |
| 1. Participant 68 | | 34 | | 48 | | **14** | | |  |  |  |  | |
| 1. Participant 70 | | 29 | | 30 | | **1** | | | **Bolded** difference scores indicate improvement. | | | | |
